# Response to Klütsch and Crapon de Caprona

**DOI:** 10.1186/1741-7007-8-120

**Published:** 2010-09-08

**Authors:** Melissa M Gray, Robert K Wayne

**Affiliations:** 1 Department of Ecology and Evolutionary Biology, University of California, Los Angeles, CA, USA

## Abstract

This article is a response to Klütsch and Crapon de Caprona

See correspondence article http://www.biomedcentral.com/1741-7007/8/119 and our original research article http://www.biomedcentral.com/1741-7007/8/16.

## Background

The origin of domestic dogs has been a source of controversy for the past two decades [1-4]. In our recent study [5], we provided evidence for an event that likely occurred early in the history of domestic dogs. We found that the Middle East is the geographic origin of all small domestic dogs. However, Klütsch and Crapon de Caprona question the conclusion from our study [5], on the basis of concerns about sample size, sampling locations, nucleotide diversity estimates and ascertainment biased single nucleotide polymorphism (SNP) data. The authors argue that these issues preclude identification of East Asia (or any other region) as the geographic origin for small domestic dogs. As discussed below, we remain confident in the findings of our study. Moreover, our results are independently supported by the archaeological record [6,7] and a recent study by vonHoldt *et al. *[8]. (Note that all figures, tables and additional files referred to throughout are those from the original paper.)

## Nucleotide diversity

Klütsch and Crapon de Caprona critique our use of nucleotide diversity estimates. They suggest this is the main support for our conclusion and that it is based on limited sequence data and samples derived from a limited region. However, we provided diverse sources of evidence for our conclusion of a Middle Eastern origin of small domestic dogs: (1) their close relationship on a principal component analysis (PCA) plot of dog-derived SNPs, (2) high levels of nucleotide diversity in insulin-like growth factor 1 gene (*IGF1*) intron 2 of Middle Eastern wolves, (3) a small number of SNP differences between haplotypes of Middle Eastern wolves and small dogs, and (4) phylogenetic proximity of the major small dog haplotype and haplotypes of Middle Eastern wolves. Although strong conclusions cannot be drawn from any single analysis, the combined results consistently point to the Middle East as the most likely origin of small domestic dogs.

We recognise that our nucleotide diversity estimates do have overlapping standard deviations, however, we do not claim that the populations are significantly different nor is this the most reliable method for determining geographic ancestry [8,9]. For more precise measures of nucleotide diversity, we refer the reader to vonHoldt *et al. *[8], which shows, on the basis of 48,000 SNPs, that Middle Eastern wolves are the dominant source of genomic variation of modern domestic dogs.

## Dog-derived SNPs

Klütsch and Crapon de Caprona argue that the dog-derived SNP dataset contains ascertainment bias that can confound our results. We acknowledged that the SNP dataset may be affected by ascertainment bias (p. 2), but we see no reason why such bias would favour high diversity in Middle Eastern over East Asian wolves since the ascertainment panel included only dogs and these were of European origin. Nonetheless, the possibility of ascertainment bias in part motivated our additional sequencing analysis. It should also be noted that the SNPs in this dataset surround *IGF1 *intron 2 with only a few SNPs in common with the sequenced region. Thus, the sequenced region is a detailed view of the area containing the diagnostic mutations for small body size.

## Sampling

Klütsch and Crapon de Caprona assert that our sample size was sparse. In our initial full-length sequencing effort, we included one to two samples from all major geographical regions (6331 bp; Figure 1, [5]) and in our secondary sequencing effort additional samples and regions were included (4811 bp) to verify sampling did not bias our results.

Furthermore, two Chinese, two Spanish, one Italian and one Indian grey wolf samples were used across all datasets. Specifically, one of the Spanish grey wolf samples used in the sequencing effort was observed to be heterozygous for two haplotypes found to be most similar to the small dog 20 SNP haplotype (Hap 50 and Hap 29; Additional file 2). Unfortunately, the Israel grey wolf samples used in the dog-derived SNP dataset were not available for sequencing. Thus, different samples from the same region were used. However, the consistency of the phylogenetic grouping of these haplotypes across sequencing experiments argues that this would not affect the outcome of our analysis. Lastly, extending the sequenced region would introduce further recombination points confounding the signal for the geographic origin of the diagnostic mutations for body size.

## South-East Asia or Spain versus a Middle Eastern origin

Overall, Klütsch and Crapon de Caprona assert that our sampling was biased in such a way as to exclude South-East Asia or Spain as an origin for small dogs. Based on mtDNA, South-East Asia has been suggested as the possible origin of dogs [1,2], but see Balloux [9], Boyko *et al. *[10], and Pang *et al. *[1] for a discussion of the limitations of mtDNA sampling. However, none of the phylogenetic analyses in our study shows a close relationship of Chinese or Spanish wolf haplotypes to the small dog haplotypes (both minor and major haplotypes). The exception is for trees constructed from the sequence data 5' of the recombination point, which show association with the minor small dog haplotype, but this region does not include the diagnostic mutations for small size. In the 6331 bp data set, all Israeli wolves fall ancestral to large dog haplotypes and cluster with the major small dog haplotype while the Chinese and Spanish wolf haplotypes cluster with the large dog haplotypes. These data also show that haplotypes from Israeli not Chinese or Spanish grey wolves have the least number of SNP differences from the small dog haplotype (3 versus 8 or 10 and 14 SNP differences, respectively). In the 4811-bp data set, all but two (out of eight haplotypes; n = 8) of the Israeli wolf haplotypes clusters with the major small dog haplotype, while the two (out of two haplotypes; n = 2) Chinese wolf haplotypes and the two (out of two haplotypes; n = 2) Spanish wolf haplotypes cluster with the large dog haplotypes. Moreover, although it is true that the Chinese wolf haplotype (Hap 4) has only three SNP differences from the minor small dog haplotype (Hap 3) in the 6331 bp dataset, these differences are found 3' of the recombination point which contain the diagnostic small dog alleles and is identical to the major small dog haplotype (Hap 5). This suggests the similarity is due to a recombination event and not ancestry with small dogs.

The authors suggest that oversampling of the Israeli wolf and undersampling of the Chinese and Spanish wolves also contributed to the exclusion of these regions as a potential source population. However, any two randomly chosen Israel samples would produce the same pattern because only one of the Israeli wolf samples was observed to be heterozygous for two haplotypes (Hap 16 and Hap 5) that both clustered with the large dogs, while both of the Chinese and Spanish wolf haplotypes clustered with large dog haplotypes. We also had similar sample sizes as China from Iran and India, which show a pattern consistent with the Israeli wolf samples and further support the Middle East as the origin of small dogs. Moreover, Hap 20 is in high frequency across Israel (4 out of 16; 0.25), Iran (1 out of 2; 0.5) and India (3 out of 4; 0.75), which clusters with the small dog haplotype. Thus if South-East Asia or any other region were the origin for the small haplotype we would expect at least one of these haplotypes to cluster with the small dog haplotype, and we find none.

## Conclusions

In conclusion, Klütsch and Crapon de Caprona critique our study by suggesting an inadequacy of sample size and location, lack of significant differences between nucleotide diversity estimates and biased results from dog-derived SNP data that all preclude identification of East Asia or any other region as a possible origin for small domestic dogs. First, we acknowledge a lack of significant differences in nucleotide diversity between populations but feel this is a minor point in light of all the other analyses in our study that support a Middle Eastern origin. Second, ascertainment bias cannot explain our results. Lastly, extensive haplotype and phylogenetic analyses and consistent patterns across samples from the Middle East all point to an origin for small dogs there. Additionally, the results of our study are independently supported by the archaeological record [6,7] and a recent study by vonHoldt *et al. *[8].

## Authors' contributions

MMG drafted the manuscript while both authors read, edited, and approved the final manuscript.

## References

[B1] Pang JF Kluetsch C Zou XJ Zhang AB Luo LY Angleby H Ardalan A Ekstrom C Skollermo A Lundeberg J Matsumura S Leitner T Zhang YP Savolainen P mtDNA data indicate a single origin for dogs south of Yangtze river, less than 16,300 years ago, from numerous wolves Mol Biol Evol 2009 26 2849 2864 10.1093/molbev/msp195 19723671 PMC2775109

[B2] Savolainen P Zhang YP Luo J Lundeberg J Leitner T Genetic evidence for an East Asian origin of domestic dogs Science 2002 298 1610 1613 10.1126/science.1073906 12446907

[B3] Vilà C Savolainen P Maldonado JE Amorim IR Rice JE Honeycutt RL Crandall KA Lundeberg J Wayne RK Multiple and ancient origins of the domestic dog Science 1997 276 1687 1689 10.1126/science.276.5319.1687 9180076

[B4] Wayne RK Leonard JA Vila C Zeder MA Genetic analysis of dog domestication Documenting Domestication: New Genetic and Archaeological Paradigms 2005 Washington DC, USA: Smithsonian Institution Press

[B5] Gray MM Sutter NB Ostrander EA Wayne RK The IGF1 small dog haplotype is derived from Middle Eastern grey wolves BMC Biol 2010 8 16 10.1186/1741-7007-8-16 20181231 PMC2837629

[B6] Davis SJM Valla FR Evidence for domestication of dog 12,000 years ago in Natufian of Israel Nature 1978 276 608 610 10.1038/276608a0

[B7] Tchernov E Valla FF Two new dogs, and other Natufian dogs, from the southern Levant J Archaeol Sci 1997 24 65 95 10.1006/jasc.1995.0096

[B8] vonHoldt BM Pollinger J Lohmueller K Han E Parker HG Quignon P Degenhardt JD Boyko A Earl DA Auton A Reynolds A Bryc K Brisbin A Knowles J Mosher DS Spady TC Elkahloun A Geffen E Pilot M Wlodzimierz J Grecco C Ettore R Bannasch D Wilton A Shearman J Musiani M Cargill M Jones PG Zuwei Q Huang W Ding Z-L Zhang Y-P Bustamante CD Ostrander EA Novembre J Wayne RK Genome-wide SNP and haplotype analysis reveals a rich history underlying dog domestication Nature 2010 464 898 903 10.1038/nature08837 20237475 PMC3494089

[B9] Balloux F Mitochondrial phylogeography: the worm in the fruit of the mitochondrial DNA tree Heredity 2010 104 419 420 10.1038/hdy.2009.122 19756036

[B10] Boyko AR Boyko RH Boyko CM Parker HG Castelhano M Corey L Degenhardt JD Auton A Hedimbi M Kityo R Ostrander EA Schoenebeck J Todhunter RJ Jones P Bustamante CD Complex population structure in African village dogs and its implications for inferring dog domestication history Proc Natl Acad Sci USA 2009 106 13903 13908 10.1073/pnas.0902129106 19666600 PMC2728993

